# The Prospective Applications of Bioelectrical Impedance Analysis in Postpartum Women

**DOI:** 10.3390/jcm14041126

**Published:** 2025-02-10

**Authors:** Julia Siek, Angelika Masiarz, Karolina Obuchowska, Monika Kopeć, Zuzanna Małysza, Żaneta Kimber-Trojnar

**Affiliations:** 1 Student’s Scientific Association at the Chair and Department of Obstetrics and Perinatology, Medical Faculty, Medical University of Lublin, 20-090 Lublin, Poland; siekjj@gmail.com (J.S.); masiarz.ang@gmail.com (A.M.); karolinaobuchowska99@gmail.com (K.O.); 2 Chair and Department of Obstetrics and Perinatology, Medical University of Lublin, 20-090 Lublin, Poland; zkimber@poczta.onet.pl; 3 Medical Faculty, Jagiellonian University Medical College, 31-501 Kraków, Poland; zuziamalysza@interia.pl

**Keywords:** bioelectrical impedance, postpartum, puerperium, breastfeeding, hydration, body composition

## Abstract

Bioelectrical impedance analysis (BIA) has a wide range of applications. For over 25 years, it has primarily been utilized for assessing body composition. This method is non-invasive, portable, widely available, cost-effective, and user-friendly, offering the advantage of repeatability and minimal dependence on patient cooperation. BIA measures the impedance of the whole body, specifically the body’s resistance to alternating current. In postpartum women, who undergo significant physiological changes following childbirth, BIA can serve as a valuable diagnostic and monitoring tool. It is commonly employed to track body weight and fat reduction, and it facilitates the differentiation of fat mass, muscle mass, and body water content. This enables the customization of nutritional plans and the development of individualized training regimens tailored to the patient’s health status. Additionally, BIA aids in the assessment of hydration status, which is particularly critical during the postpartum period when women often experience fluid retention. Furthermore, optimal hydration is essential for lactation and maintaining favorable conditions for breastfeeding. BIA is also invaluable for evaluating nutritional status, micronutrient balance, and preventing both overweight and malnutrition. Moreover, BIA supports physical recovery by monitoring muscle mass, thereby assisting in the assessment of pelvic floor muscle regeneration following childbirth.

## 1. Introduction

### 1.1. Puerperium

During the postpartum period, also known as the puerperium, the physiological changes associated with pregnancy return to a non-pregnant state. This is a particularly difficult time in mothers’ lives as they face many new challenges, including feeding and caring for a newborn, physical recovery, hormonal changes, and sleep disruptions [1]. Already in 1975, Sheila Kitzinger stated that the postpartum period should be referred to as the “fourth trimester”, which cannot be neglected by both medical care and the patient and her family [2].

The changes that occur during pregnancy, supporting both the development of insulin resistance and the increase in the amount of adipose tissue, can be a so-called “double-edged sword” [3]. Weight gain and metabolic changes that manifest during pregnancy, if not managed, can have negative consequences for the woman after childbirth, increasing the risk of long-term complications [4]. Current literature emphasizes that the early postpartum period, i.e., the period during postpartum hospitalization, allows for early identification of women at significant risk of developing lifestyle diseases. This is particularly important in the case of patients with disorders related to the placenta, such as fetal growth retardation, preeclampsia or placental abruption, as well as in patients with gestational diabetes mellitus (GDM), who require counseling and constant care due to increased risk of developing cardiovascular disease and diabetes mellitus.

The postpartum period begins at the time of birth of the newborn. Its end is less precisely defined but is usually considered to occur at six weeks after delivery because the effects of pregnancy on many systems during this time largely return to their pre-pregnancy state [5].

The average weight loss following the expulsion of the fetus, placenta, and amniotic fluid is approximately 6 kg. Immediately after the delivery of the placenta, the uterus initiates the process of returning to its pre-pregnancy state, referred to as uterine involution. The uterine weight decreases from about 1 kg immediately after delivery to 60 g within six to eight weeks. Normal abdominal wall muscle tone usually returns within a few weeks. However, separation of the rectus abdominis muscles may persist longer, which may be associated with abdominal discomfort. Continued uterine contractions, together with loss of lochial fluid and excess intra- and extracellular fluid, result in an additional weight loss of 2 to 7 kg during the postpartum period. Approximately half of the weight gained during pregnancy is lost within the first six weeks after delivery, with a slower rate of weight loss observed in the next six months after delivery [6].

Physiological changes in the cardiovascular system are particularly significant in patients with pre-existing cardiac conditions. Within the first 10 min following a term vaginal delivery, both cardiac output and stroke volume increase by approximately 60% and 70%, respectively. At one hour postpartum, both cardiac output and stroke volume remain elevated (by approximately 50% and 70%, respectively), while heart rate decreases by 15%. Blood pressure remains stable. These increases in stroke volume and cardiac output are most likely attributable to improved cardiac preload due to autotransfusion of uteroplacental blood into the intravascular space. As the uterus decompresses post-delivery, a reduction in mechanical compression of the vena cava further facilitates increases in preload. A study evaluating cardiac output and stroke volume in 15 healthy, non-laboring patients at 38 weeks of gestation and again at 2, 6, 12, and 24 weeks postpartum showed a gradual decrease in cardiac output from 7.42 L/min at 38 weeks of gestation to 4.96 L/min at 24 weeks postpartum [7]. Notably, by two weeks postpartum, there were significant reductions in left ventricular size and contractility when compared to term pregnancies. In patients with antepartum or intrapartum preeclampsia, blood pressure typically decreases within 48 h after birth but may rise again three to six days postpartum.

Pregnancy-related hematologic changes typically return to baseline within 6 to 12 weeks postpartum. Within this period, the rate and pattern of resolution of pregnancy-related alterations in specific hematological parameters vary. Of particular importance, the prothrombotic state persists for several weeks, resulting in an increased risk for thromboembolic disease in the postpartum period. Thromboembolic events represent the leading cause of direct maternal mortality globally, particularly in high-income countries. Venous thromboembolism (VTE) occurs more frequently during pregnancy, more often in the postpartum period than during pregnancy, and more frequently following cesarean delivery than after vaginal birth [8,9]. The risk of VTE is highest in the first few weeks postpartum, gradually decreasing to baseline levels by 12 weeks after delivery [10].

Following delivery, several hormonal changes occur. The concentration of human chorionic gonadotropin (hCG) decreases and typically becomes undetectable by the 14th day postpartum [11,12,13]. Postpartum hot flashes, which are relatively common, are associated with thermoregulatory dysfunction [14], originating at the hypothalamic level due to a rapid decline in estrogen following placental expulsion. The state of hyperprolactinemia induced by breastfeeding further diminishes estrogen production. Gonadotropins and sex steroids remain at low levels during the first two to three weeks post-delivery. The extent to which breastfeeding inhibits the secretion of the gonadotropin-releasing hormone (GnRH) is influenced by the intensity of breastfeeding, as well as the mother’s nutritional status and body weight [15,16,17]. Lactation imposes an energy demand on metabolism. When maternal nutrition is adequate and body weight and composition are normal, intensive lactation is less likely to cause prolonged GnRH inhibition [18,19]. Conversely, when nutrition is insufficient to meet the energy requirements for both daily activities and lactation, GnRH suppression is more likely to persist, leading to prolonged oligo- or anovulation. Among women exclusively breastfeeding, approximately 40% will remain amenorrheic for six months postpartum [20,21]. Postpartum amenorrhea during breastfeeding may be partly attributed to higher prolactin levels compared to women who ovulate during breastfeeding, as prolactin inhibits the pulsatile release of GnRH from the hypothalamus [22].

An expert panel convened by the World Health Organization concurred with most published guidelines, recommending that a mother and her healthy, full-term infant, following an uncomplicated delivery, be monitored by a qualified healthcare professional for 24 to 48 h postpartum [23]. Despite educational efforts, many patients report difficulty distinguishing normal postpartum symptoms from complications and feel inadequately prepared to manage the postpartum experience, including physical recovery, breastfeeding, infant care, and issues such as depression and anxiety [24]. Xia et al., in a meta-analysis published in 2022, proved that breastfeeding significantly reduces the risk of postpartum depression. Breastfeeding for more than 1 month was associated with a 37% lower risk of postpartum depression [25]. On the other hand, the results of another meta-analysis showed the great importance of general well-being, positive emotions, and life satisfaction for patients’ recovery and convalescence [26].

One of the most important public health priorities is the promotion and support of breastfeeding among mothers. Breastfeeding provides health benefits to both infants and mothers. Continuing to breastfeed, even if not exclusively for the infant, still offers health benefits to both. Support for breastfeeding from healthcare providers has been associated with higher rates of breastfeeding after hospital discharge [27].

Another important aspect for the mother after delivery is returning to her pre-pregnancy weight. There is currently no evidence to support specific recommendations for physical activity after delivery [28]. The primary role here is played by an appropriate diet and breastfeeding. Promoting healthy eating, physical activity, maintaining or achieving a healthy body mass index (BMI), healthy sleep patterns, and smoking cessation among smokers at the postnatal visit can help reduce the risk of future cardiovascular disease. Women diagnosed with GDM during pregnancy should undergo an oral glucose tolerance test after delivery. They should be counseled about the increased risk of future diabetes, even if their postnatal glucose tolerance test results are normal. These individuals may benefit significantly from lifestyle interventions such as weight loss, especially if they are overweight or obese. Mothers who have had GDM, preeclampsia, or preterm birth are at increased risk of future cardiovascular disease, even if their postnatal blood pressure and glucose tolerance test results are normal. The American College of Obstetricians and Gynecologists recommends that physicians counsel women with these pregnancy-related complications about their increased risk of cardiovascular disease [29]. The American Heart Association recommends monitoring cardiovascular risk factors during routine preconception and inter-conception visits, including screening for factors such as blood pressure, fasting lipid panel, body weight, and glucose intolerance or diabetes, as well as considering other less commonly recognized biomarkers [30]. Increased awareness of cardiovascular risk may increase the motivation of individuals to mitigate modifiable risk factors by adopting a healthy lifestyle.

Given the above issues resulting from both the physiological course of the postpartum period and the superimposition of metabolic disturbances, both preexisting and developing during pregnancy, the topic of the current narrative review of the use of bioelectrical impedance in postpartum women seems worthy of presentation.

### 1.2. Bioelectrical Impedance Analysis

The development of advanced functional exploration tools in medicine has revolutionized the diagnosis of various pathologies and significantly improved patient care. Body composition measurement techniques are continuously evolving, leading to greater precision and ease of measurement [31,32]. While the BMI provides valuable information, it is less informative than body composition analysis. Body weight refers to the total mass of all tissues in the body, whereas body composition assessment involves measuring body mass, total body water, and the relative proportions of fat and lean mass. Although body composition evaluations are primarily conducted for research purposes, they hold significant clinical importance in specific contexts, such as identifying individuals with excessive visceral fat who are at increased risk of cardiovascular diseases and diabetes. Other clinical indications include the assessment of malnourished patients, those with edema (where excess fluid contributes to increased body weight), and individuals whose body composition has been altered due to factors such as aging, diseases, or hormonal imbalances [33,34,35,36,37,38,39,40].

Various techniques for assessing body composition include Dual-Energy X-ray Absorptiometry (DEXA), BIA, Computed Tomography (CT), Magnetic Resonance Imaging (MRI), Nuclear Magnetic Resonance Spectroscopy, isotope-based body water measurements, and water or air displacement methods such as hydrodensitometry or whole-body plethysmography. Currently, DEXA is considered the gold standard for body composition measurement. However, BIA is the most commonly used method due to its numerous advantages, including non-invasiveness, lack of radiation exposure, cost-effectiveness, convenience, and ease of execution.

The scientific basis for BIA stems from the work of George Simon Ohm (1788–1854). The clinical applications of this method gained attention in the 1960s when French physician Thomasset conducted studies on impedance measurements in clinical medicine [41]. In the 1990s, researchers such as Lukaski, Kushner, Segal, and Scholler proposed the “five-compartment model,” describing the human body in terms of five segments: the right arm, left arm, torso, right leg, and left leg. To validate the accuracy of BIA, these scientists developed equations for intracellular and extracellular water, total body water, and impedance indices. Following extensive research on nutritional status, the National Institutes of Health (NIH) officially recognized the clinical value of BIA in 1994, thereby allowing its use in clinical practice [42].

BIA is a simple and widely used technique, typically involving four disposable electrodes placed on one arm and one leg [43,44]. Proper electrode placement is crucial, as even minor deviations can result in significant errors in impedance measurement, which in turn affect the accuracy of body water content estimates. When using BIA devices, it is essential to apply validated equations to estimate the percentage of body fat [45]. BIA measures the electrical impedance, or resistance, of tissues through which a low-intensity current (<1 mA) passes [46]. The results of a BIA test are influenced by proper preparation; for accurate measurements, it is recommended that the last meal be consumed at least four hours before the test, alcohol consumption should be avoided for at least 48 h, the bladder must be emptied 30 min prior to the test, and intense physical activity should be avoided for at least 12 h before the test. Factors such as gender, age, maturity, kidney disease, ambient temperature, and the time of day can also affect BIA measurements [47,48,49].

Due to their straightforward procedure, rapid results, and safety, BIA-equipped devices have become widely accessible. This method is now widely applied in medicine to assess total body water (TBW), fat, and skeletal muscle mass. BIA is useful for monitoring changes in body composition in various chronic conditions, such as heart failure, obesity, diabetes, kidney disease, and cancers. Further research has applied BIA to cases involving fatty liver, ischemic stroke, sarcopenia, spina bifida, and malnutrition [44,48,49,50,51,52].

**Figure 1:**
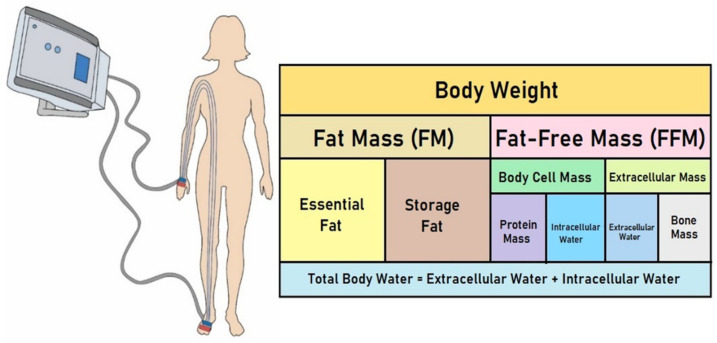
Body composition assessment using a four-electrode BIA device.

BIA most commonly involves generating a continuous low-intensity electrical current (≤1 mA; 50 kHz) through two outer electrodes and measuring the voltage through two other (inner) electrodes placed on the upper and lower extremities on the same side of the body [51]. Impedance corresponds to the resistance offered by the tissue, a biological conductor, to the flow of low-intensity alternating current [53]. Impedance is a function of body water content and the frequency of the applied alternating current. The basic principle of BIA is to treat the human body as a conducting cylinder, use the electrical properties of the internal and external fluids and the cell membrane, measure resistance (R) and reactance (XC) at different electrical frequencies, and estimate body composition using empirical regression equations [49]. These equations estimate parameters such as TBW, intracellular and extracellular water (ICW and ECW), lean tissue mass (LTM), adipose tissue mass (ATM), and body cell mass (BCM) (Figure 1).

R is primarily determined by the electrical properties of intracellular and extracellular fluids, while ICW and ECW can be calculated accordingly. XC is mainly determined by the capacitive properties of the cell membrane and reflects the somatic cell population. The phase angle (PhA) is an indicator of the relationship between XC and R, which can reflect the integrity of membrane structure and function. Thus, BIA enables a detailed analysis of body composition and hydration status by assessing the proportion of fat mass (FM) and fat-free mass (FFM), TBW, basal metabolic rate (BMR), and estimated average energy requirement (EAR) [51]. Moreover, the interpretation of body hydration status depends on factors such as intracellular and extracellular hydration states and the presence of third-space fluid in cases of edema [51].

Currently, the most commonly used techniques include multi-frequency BIA (MF-BIA) based on the three-compartment (3-C) model, which distinguishes overhydration (OH), ATM, and LTM [49].

In summary, BIA is a non-invasive, cost-effective, and portable method for estimating body composition [53]. While the accuracy of BIA body composition measurement is still debated, it remains widely used in clinical and research settings. Numerous studies support its significant role in nutritional management and disease prediction. Body composition measurement via BIA not only provides a better understanding of the pathophysiology of many diseases but also, in some cases, helps track their progression to guide treatment strategies.

It appears that BIA could become an invaluable diagnostic tool in the postpartum period when women are more susceptible to dehydration and excessive gestational weight gain (EGWG).

Our study aims to evaluate the potential relevance of BIA in monitoring the health status of women in the puerperium.

## 2. Materials and Methods

We conducted a comprehensive literature review utilizing the Web of Science, PubMed, and Scopus databases, considering articles published in English up to 31 December 2024. Inclusion criteria encompassed randomized clinical trials, meta-analyses, systematic reviews, human studies, and recommendations from scientific societies. Exclusion criteria comprised duplicate articles, case reports, conference abstracts, commentaries, and articles published in languages other than English. The following keywords were used: “bioelectrical impedance”, “postpartum”, “post-partum”, “puerperium”, “breastfeeding”, “lactation”, “hydration”, “electrolytes imbalance”, “edema”, “postpartum infection”, “postpartum sepsis”, “body composition”, “rehabilitation”, “regeneration”, and their combinations, where “bioelectrical impedance” was associated with one of three terms: “postpartum”, “post-partum”, or “puerperium”, and one of the remaining terms.

The use of a mixed-method approach to collecting relevant literature is common in systematic reviews, especially when the topic is relatively narrow and rare. To minimize bias, we tried to adopt clear inclusion and exclusion criteria and document the search process, including sources and search terms used. Cited papers were selected and reviewed by at least two independent reviewers to provide an objective approach to the topic under review.

## 3. Results

**Figure 2:**
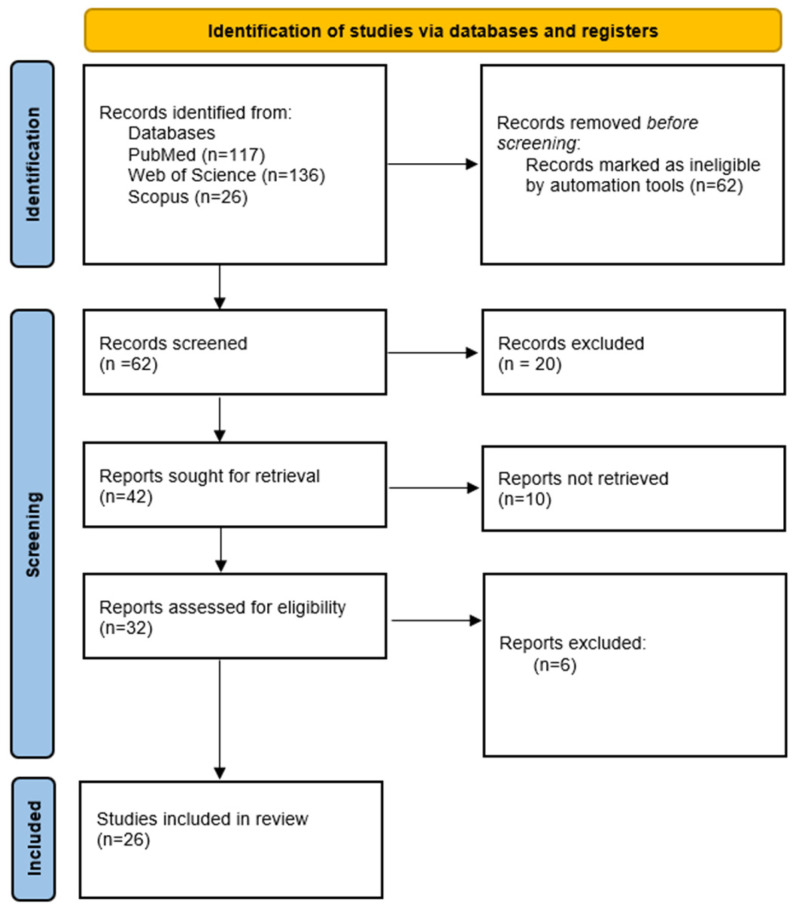
Study selection for bioelectrical impedance as a way to monitor changes in the body composition of women in postpartum.

**Figure 3:**
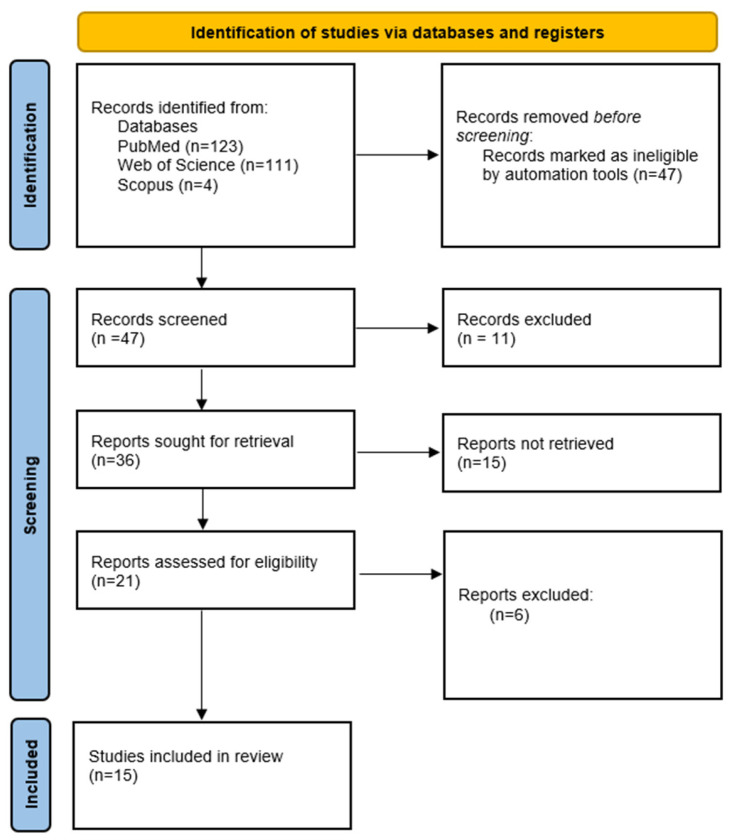
Study selection for bioelectrical impedance and assessment of hydration status in women in puerperium.

**Figure 4:**
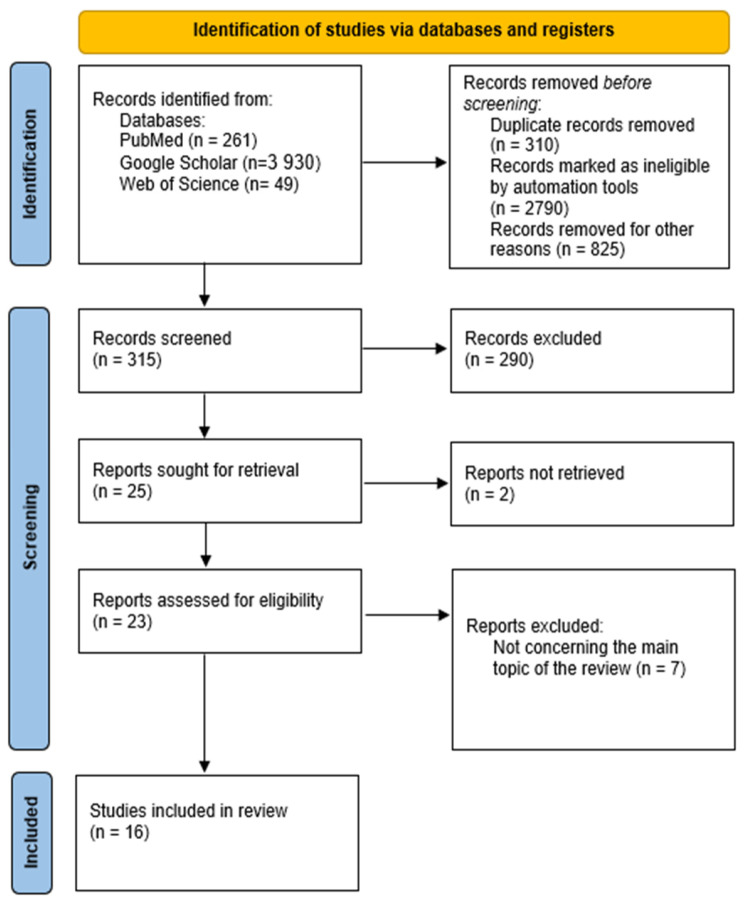
Study selection for sufficiency of bioelectrical impedance in monitoring postpartum complications—diagnosis of edema, electrolyte imbalances, infections, and preeclampsia.

**Figure 5:**
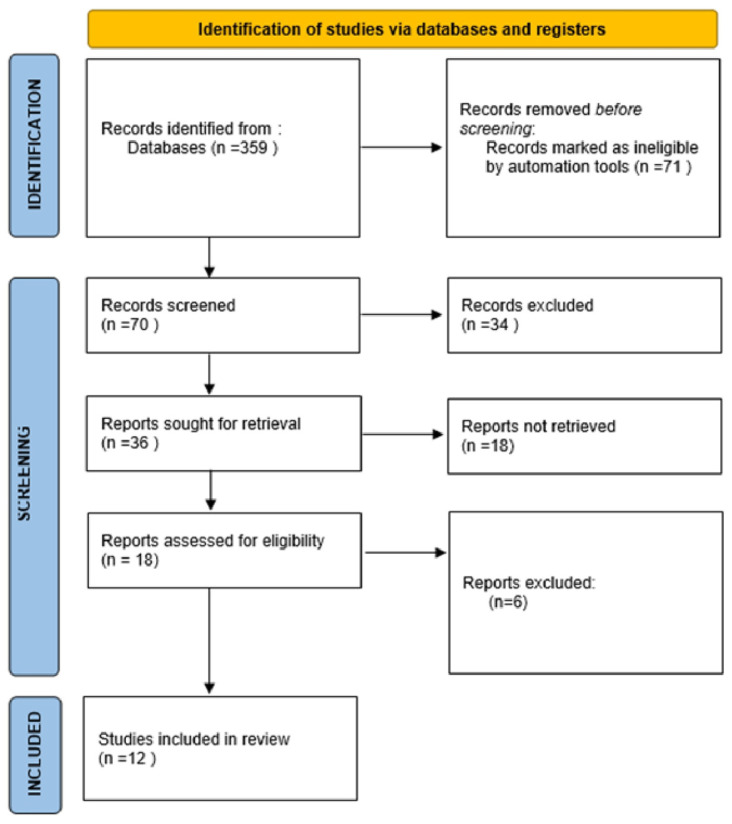
Study selection for application of bioelectrical impedance in the rehabilitation of postpartum women.

Primarily, we aimed to adhere to the PRISMA guidelines in our review. However, we also intended to discuss various aspects of BIA application in postpartum women and to extend the topic to encompass other potential applications of this method based on studies conducted in non-postpartum populations. Consequently, in the following subsections, we followed the PRISMA guidelines while describing different possibilities for BIA use in postpartum women. As a result, we created four flow diagrams to illustrate our search strategies (Figure 2, Figure 3, Figure 4 and Figure 5).

## 4. Bioelectric Impedance as a Way to Monitor Changes in the Body Composition of Women in Postpartum

Maternal body weight during pregnancy encompasses various components, including the products of conception (i.e., fetus, placenta, and amniotic fluid), uterine and mammary tissues, maternal body water (intracellular and extracellular), and adipose tissue. Over the course of pregnancy, these components undergo significant adaptive changes in varying amounts, which can substantially influence the interpretation of individual weight gain [54,55]. After delivery, each component of body composition evolves in distinct ways and quantities. Consequently, the relative distribution of these components concerning postpartum weight retention can vary. Therefore, routine measurements of body weight may not accurately reflect changes in body composition and may fail to provide meaningful insights into the factors influencing postpartum weight retention [56].

The desired weight reduction should primarily involve a loss of FM, with minimal changes in FFM and body fluid components. Substantial losses in FFM and water components are often associated with muscle weakness and functional impairments [57,58,59].

The study conducted by Cho et al. [56] used BIA to assess body composition at three postpartum time points: 2 days, 2 weeks, and 6 weeks after delivery. The results indicated that FM and visceral fat increased postpartum, with visceral fat showing a significant 45% increase. This marked increase represents the most substantial change in body composition and reflects postpartum fat mobilization in central areas. In individuals with obesity, the ECW/ICW ratio is typically higher compared to those of normal weight due to the distribution of adipose tissue primarily in extracellular spaces [57]. Thus, postpartum FM increases should be correlated with a rise in the ECW/ICW ratio. However, the study observed a slight decrease in the ECW/ICW ratio from 0.66 to 0.62, though values remained within the normal range. This reduction may be attributed to more significant changes in ECW compared to ICW, suggesting that the postpartum phase involves distinct hemodynamic and fluid balance mechanisms [56].

Postpartum weight normalization is strongly recommended. However, studies indicate that approximately 75% of women do not achieve their pre-pregnancy weight within the year following childbirth [60]. Breastfeeding has been shown to influence maternal weight loss. Jayasinghe et al. established a correlation between extended breastfeeding duration and enhanced maternal weight loss post-delivery, substantiating lactation as an effective weight reduction strategy. The process of breastfeeding, along with the associated energy expenditure of milk production, promotes lipolysis and mobilizes fat reserves accumulated during pregnancy, contributing to a reduced rate of postpartum weight retention [61,62].

While body composition is influenced by lifestyle factors such as diet and exercise in the general population, limited research has explored the relationship between lifestyle and body composition specifically during the postpartum period. Therefore, incorporating a tailored dietary plan with regular body composition assessments may support healthier and more regulated weight loss during the postpartum phase. Interventions promoting healthy diet and physical activity behaviors in the postpartum period may reduce weight retention and decrease obesity-related risks in subsequent pregnancies [63,64].

## 5. Bioelectrical Impedance and Assessment of Hydration Status in Postpartum Women

Water is an essential nutrient for human survival and development as it regulates osmotic pressure, maintains electrolyte balance, and controls body temperature. Hydration is determined by the equilibrium of water intake and output. Furthermore, proper hydration is critical for the maintenance of normal physical and cognitive functions [65,66].

**Figure 6:**
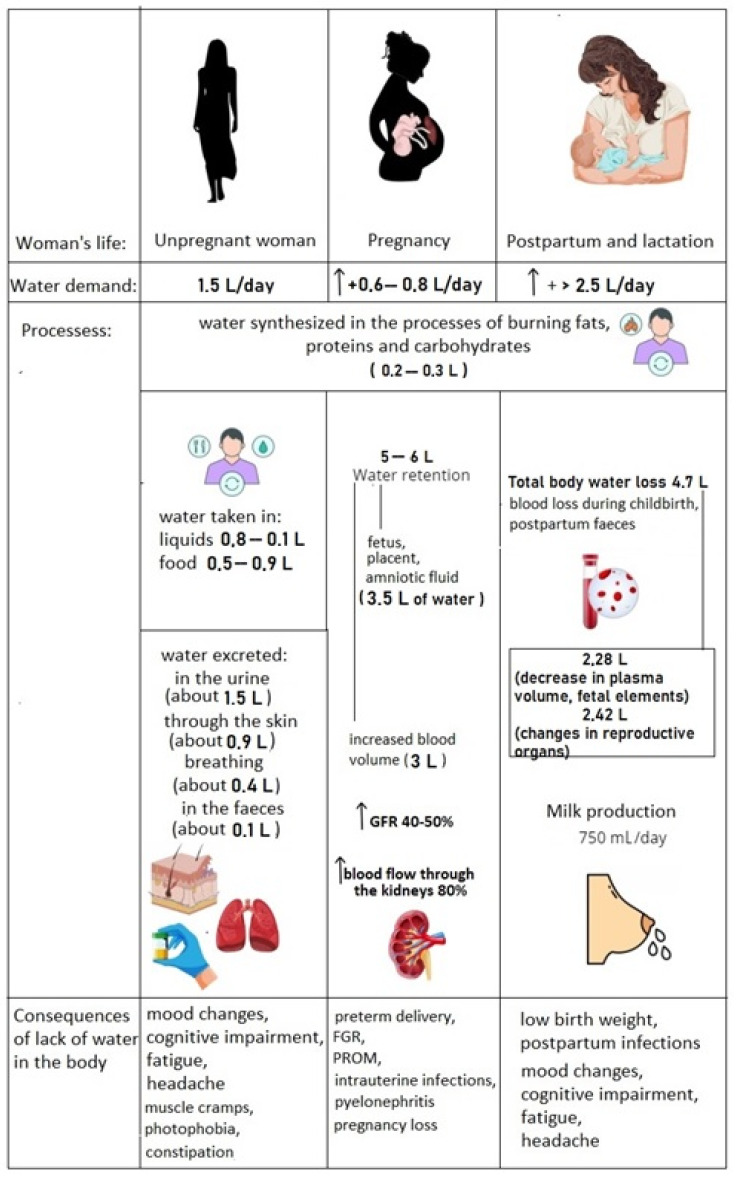
Water management in non-pregnant, pregnant and breastfeeding women.

During pregnancy, women experience significant physiological changes, including a 20% increase in total body water (approximately 7 L), which plays a major role in the typical gestational weight gain of 12 kg at delivery [67]. Among its various functions, this increased body water helps maintain the volume of amniotic fluid, aids in expanding plasma volume, and constitutes a significant portion of the growing fetus [68] (Figure 6).

Research on the variations in the postpartum ECW/ICW ratio over time is limited. It has been established that obese individuals possess higher ECW/ICW ratios compared to those of normal weight, a phenomenon attributed to the distribution of adipose tissue fluids, which are primarily located in extracellular spaces [57].

In the study by Cho et al. [56], BIA was used to measure body composition at three postpartum time points: 2 days, 2 weeks, and 6 weeks after delivery. ECW is composed of interstitial fluid and plasma volume. Plasma volume decreases immediately after delivery, leading to a reduction in ECW. Plasma volume during the puerperium remained 10–15% higher than non-pregnancy levels at 3 weeks postpartum but normalized to pre-pregnancy levels by approximately 6 weeks. In this study, the mean ECW reduction from 2 days to 6 weeks postpartum was 2.28 ± 1.02 kg, representing a 16.12% reduction. ICW during pregnancy likely reflects physiological changes in the mother’s body, such as the growth of breast and uterine tissues [69]. Peak ICW growth at the end of the third trimester was correlated with swelling of tissues like the breasts and pelvis, which are involved in preparations for childbirth and puerperium. Both the vagina and uterus shrink to pre-pregnancy size around 4 and 6 weeks postpartum, respectively [55,56]. Cho et al. reported a mean ICW reduction of 2.42 ± 1.29 kg, reflecting an 11.32% decrease from 2 days postpartum. Consequently, the reduction in TBW was 4.70 ± 2.24 kg, which contributed to the observed postpartum weight loss [56].

Human milk provides optimal nutrition for infants, offering a balanced array of essential nutrients and bioactive components. Furthermore, exclusive breastfeeding for the first six months is associated with reduced risks of infections, chronic diseases, obesity, and type 2 diabetes later in life [62,70].

Hydration is vital for the lactation process, as water is a key component of breast milk. Adequate hydration influences both the quantity and quality of milk produced and also impacts the mother’s overall health. Water constitutes approximately 87% of breast milk [71]. Throughout lactation, milk output gradually increases, with an average of 750 mL per day by 6 weeks postpartum, resulting in a significant extra loss of water [70]. Bzikowska-Jura et al., using BIA, found a high correlation between maternal body composition and the total protein concentration in milk. Specifically, a positive correlation was observed with maternal body weight, BMI, and fat mass percentage, while a negative correlation was found with the percentage of total body water during the third month of lactation. Lower total body water content was characteristic of women with higher body fat percentages [70]. Hydration affects not only milk quantity but also its quality. Adequate fluid intake supports the delivery of essential nutrients to the milk, including vitamins, minerals, and proteins, which are vital for the proper development of the infant. Insufficient hydration may result in reduced concentrations of these components in breast milk [72]. Quinn et al. and Kugananthan et al. observed that higher fat mass percentages correlated with increased protein content in milk [73,74]. These findings suggest that the nutritional quality of human milk is not solely determined by maternal diet but is also contingent on maternal body composition.

Proper hydration aids in maintaining electrolyte balance in the mother’s body, which is crucial for the proper functioning of metabolic processes, including milk production [72]. Water is involved in the transport of nutrients to tissues, which is particularly important in lactation. Lactation is associated with heat release, which may cause a feeling of overheating. Water also plays a role in regulating body temperature. To maintain adequate hydration, breastfeeding mothers should consume sufficient fluids throughout the day. It is generally recommended that women drink at least 2.5 L of water per day; however, during lactation, this requirement may be higher. In addition to water, fluid balance can be supported by other beverages, such as herbal teas (e.g., nettle, which supports lactation) or isotonic drinks, which help maintain appropriate electrolyte levels [75]. Fluid needs also depend on lifestyle factors, ambient temperature, physical activity, and individual body requirements. In hot weather or during periods of intensive breastfeeding or physical exertion, fluid demand may increase. To produce an adequate amount of milk, the mother’s body must remain well-hydrated. A deficiency of water can lead to a decrease in milk volume, which will negatively affect the ability to feed the child [72].

Undoubtedly, BIA measurements during the puerperium have certain limitations. Factors such as changes in hydration and the balance of water and electrolytes can influence the measurement parameters [76,77]. During the puerperium, elevated levels of vestibular natriuretic peptide in the serum inhibit aldosterone, angiotensin II, and vasopressin, promoting sodium excretion in the urine, which in turn affects the water-electrolyte balance [78]. It is suggested that further monitoring of changes in fat mass (FM) and fat-free mass (FFM) may be important once fluid distribution stabilizes [79].

## 6. Sufficiency of Bioelectrical Impedance in Monitoring Postpartum Complications—Diagnosis of Edema, Electrolyte Imbalances, Infections, and Preeclampsia

Childbirth does not signify the cessation of risks for women. The postpartum period can also be fraught with complications.

Postpartum infections can affect the urinary tract, uterus, or surgical sites following cesarean sections. Infections occur in 5–7% of postpartum women, with higher rates observed among those undergoing cesarean deliveries [80]. The potential of bioelectrical impedance analysis (BIA) to diagnose infections in general medical settings has been explored. For example, phase angle measurements derived from BIA have been investigated as predictors of infection risk and indicators of cellular health. Specifically, studies have shown that individuals with infections during the postoperative period exhibited significantly lower standardized phase angle (SPA) values the day before surgery compared to those without infections. However, there is a lack of specific research on the application of BIA in the diagnosis of postpartum infections [81]. Puerperal sepsis is one of the leading causes of maternal mortality globally, accounting for 10–15% of postpartum deaths [82]. In the context of sepsis, accurate assessment of fluid status is crucial for effective management. A study investigating the relationship between the need for mechanical ventilation and BIA vector length (VL) in sepsis patients found that, after accounting for age, sex, and severity of illness, a shorter VL, associated with a hypervolemic state, predicted continued reliance on invasive mechanical ventilation. Conversely, individuals with a longer VL were 30% more likely to avoid the need for such interventions. These findings suggest that BIA may assist clinicians in making informed decisions by providing valuable insights into a patient’s fluid status [83]. Another study, based in Seoul, demonstrated that BIA measurements could be obtained in under three minutes with minimal inter-operator variability and no invasive procedures. This study revealed significant differences in ECW/TBW and ICW/TBW ratios between survivors and non-survivors of sepsis. The researchers determined that intracellular dehydration, coupled with extracellular edema, was characteristic of non-survivors following fluid resuscitation. The study suggested that phase angle, TBW, ICW, and ECW could be promising indicators for evaluating body water distribution during diagnosis and fluid treatment [84]. However, a different study assessing the validity of BIA for fluid measurement in patients with septic shock found that while BIA did not exhibit systematic errors or biases, it had wide limits of agreement, indicating substantial random errors. This led the authors to conclude that BIA is not sufficiently accurate for guiding fluid management in septic shock patients [85]. Although specific research on the use of BIA in postpartum sepsis is limited, insights from broader sepsis studies can be useful.

The reliability and precision of BIA in the postpartum population may be further compromised due to physiological changes in fluid distribution that occur during this phase. A case report on a 25-year-old pregnant woman with chronic kidney disease demonstrated that serial bioimpedance measurements provided valuable information in distinguishing between normal pregnancy weight gain and overhydration. Segmental bioimpedance allowed for more precise modifications to dry weight targets, accounting for the increase in amniotic fluid and fluid retention in the legs as gestational age progressed [86]. BIA has also been shown to differentiate patients with preeclampsia from those with pregnancy-induced hypertension, as well as to identify individuals at risk of small-for-gestational-age fetuses earlier in pregnancy [87]. In a study assessing maternal body fluid composition in uncomplicated versus hypertensive pregnancies, it was found that late-onset preeclampsia (LPE) was associated with higher TBW and ECW compared to uncomplicated pregnancies. Additionally, the ECW/ICW ratio was elevated in preeclamptic patients compared to those with uncomplicated pregnancies or gestational hypertension [88]. Since maternal vascular maladaptation is more pronounced in early-onset preeclampsia (EPE) than in LPE, the increased TBW and ECW in LPE may indicate fluid overload in a venous system that is relatively well adapted [89]. A study published in 2010 examined BIA parameters in preeclamptic women, both directly (resistance and reactance) and indirectly (ICW and ECW), and found that preeclamptic women had higher TBW and lower resistance and reactance values compared to healthy pregnant women. These findings suggest that BIA can differentiate between preeclamptic and healthy pregnancies and may provide insights into the mechanisms underlying preeclampsia [90]. Other researchers have noted that BIA could serve as an adjunct test in evaluating body composition and hydration status, both of which are relevant to pregnancy complications [44]. Bioimpedance cardiography, a similar technology, has also been explored in pregnancy. A study published in 2016 suggested that bioimpedance cardiography could provide additional information for patients at risk for preeclampsia despite different hemodynamic patterns [91]. Hemodynamic profiles in pregnancy-related hypertensive disorders were examined in another study, which supported the use of bioimpedance in tracking alterations in hemodynamic patterns throughout different stages of preeclampsia [92].

While BIA can provide valuable insights into hydration status, it is not sufficient for diagnosing specific electrolyte imbalances due to its limitations. BIA estimates body composition based on the resistance and reactance of body tissues to electrical currents but does not directly measure individual electrolyte concentrations, such as sodium or potassium levels, which are critical for diagnosing electrolyte imbalances [93]. BIA measurements may be influenced by changes in electrolyte and hydration levels independently, which could distort the results. Thus, although BIA is capable of identifying variations in body water content, it cannot determine whether these variations are caused by electrolyte abnormalities [94]. While BIA remains a valuable tool for assessing overall body composition and hydration status, accurate diagnosis of electrolyte imbalances requires direct measurement of electrolyte levels through blood tests and clinical evaluation.

## 7. Application of Bioelectrical Impedance in the Rehabilitation of Postpartum Women

BIA is gaining increasing popularity in medical fields, including the rehabilitation of postpartum women, due to its ability to monitor the progress of postpartum recovery, particularly in the context of abdominal and pelvic floor muscle regeneration and the analysis of metabolic changes. One of the reasons for its growing use in evaluating postpartum body composition is its relatively lower cost compared to other methods, such as MRI or CT, its wide availability, and its ease of use [95,96,97,98].

During the postpartum period, women often observe significant changes in their bodies. These changes are primarily influenced by physiological and hormonal factors, which drive alterations in body composition. These include, but are not limited to, water loss, reduced blood volume, decreased hormone levels, and the recovery of internal organs, including the gradual return to a pre-pregnancy state. Bioelectrical impedance measurement during this period can aid in tracking progress in postpartum rehabilitation, particularly regarding changes in body composition related to abdominal and pelvic floor muscles. After childbirth, women may experience weakness in these areas due to tissue stretching, which impairs their function. The BIA method facilitates the measurement of both muscle and fat mass. According to Garr Barry et al., weight retention after childbirth is a significant contributing factor to future obesity and the risk of chronic diseases [96]. The growing interest in bioelectrical impedance can be attributed to evidence suggesting that postpartum fat mass and fat-free mass are more critical predictors of future health risks than body weight or BMI [96].

Following delivery, women may experience a loss of body weight, which is linked to the loss of water and blood, although the reduction of fat and muscle tissue is also significant. Lukaski et al. demonstrated in their studies on hydration changes in pregnant and postpartum women using bioelectrical impedance that body weight post-delivery was significantly lower compared to the third trimester but still considerably higher than pre-pregnancy levels [54]. In terms of hydration, total body water (TBW) significantly decreased post-delivery relative to third trimester values, though it remained unchanged from pre-pregnancy levels [54]. Hematocrit levels also increased post-delivery compared to pre-pregnancy values [54].

BIA enables the monitoring of changes in muscle and fat mass, which is of considerable importance in evaluating the effectiveness of rehabilitation exercises. This reflects how a woman’s body adapts to the post-delivery period. Normal body fat content for women ranges between 15–20%, muscle tissue is ideally maintained at around 30%, water content at 55%, and calf mass at 12–13.5% [99]. An increase in muscle mass suggests improved muscle strength and regained fitness. Furthermore, BIA is invaluable for monitoring the volume and function of pelvic floor muscles, which are often weakened after childbirth. Regular measurements can assess improvements in pelvic floor muscle function, providing insights into the degree of recovery. In the postpartum period, particularly following a cesarean section, a woman’s body undergoes various regenerative processes, including the reconstruction of abdominal muscles, the pelvic floor, the perineum, and other affected areas. Muscles possess specific electrical properties that differ from other tissues, and BIA helps detect muscle weakness, which is especially important in the postpartum phase.

Additionally, BIA aids in the assessment of water and electrolyte balance. Postpartum women often experience water retention due to hormonal changes, such as a decrease in progesterone levels, which has a diuretic effect. Postpartum, it is crucial to restore balance, and BIA helps evaluate body water levels, offering insights into hydration and tissue health, including muscle condition. It also helps detect excessive water accumulation or dehydration, both of which can impede recovery. This is significant because water deficiency may slow down regenerative processes. Electrical conductivity correlates with the amount of water or electrolytes [44]. High-frequency currents penetrate all body water, while low-frequency currents only affect ECW [44]. Using bioelectrical impedance, it is possible to measure both ICW and ECW, as well as assess fat and muscle mass [44].

BIA also enables the analysis of metabolic changes in postpartum women, which is of considerable importance for understanding the recovery process and regaining strength post-delivery. This method allows for the evaluation of metabolic rate, providing insights into the basal metabolic rate, which indicates the rate at which the body burns calories at rest [98]. This is crucial for managing body weight postpartum and optimizing the recovery process. BIA further supports the monitoring of rehabilitation progress. Detailed analysis of body composition changes, such as increased muscle mass and simultaneous fat loss, can indicate successful rehabilitation and recovery.

Assessing the relationship between a woman’s body composition and energy balance can help create a personalized diet plan [44]. Additionally, BIA allows for the evaluation of changes in fat distribution. Postpartum, excessive fat accumulation, particularly in the abdominal region, is common due to hormonal changes. As fat mass increases, electrical impedance also rises. BIA allows for the monitoring of whether these changes reverse during rehabilitation, potentially indicating the effectiveness of the therapy used. BIA results can guide the personalization of exercise plans aimed at improving abdominal and pelvic floor muscle strength while reducing fat mass [56]. If the desired changes do not occur, the exercise program can be modified. A tailored diet plan can also be prescribed to support muscle regeneration and restore metabolic balance, aiding the process of returning to full fitness after childbirth [100]. Moreover, BIA helps assess the impact of hormonal changes on metabolism, body weight, and recovery. Observing these changes in conjunction with other diagnostic tools can enhance comprehensive care for postpartum women.

Incorporating BIA into postpartum care enables the development of personalized exercise programs. By assessing parameters such as fat mass and fat-free mass, healthcare providers can recommend specific physical activities aimed at promoting healthy weight loss and enhancing overall fitness during the puerperium. A study evaluating an interdisciplinary lifestyle and psychosocial intervention for women with GDM demonstrated that such tailored approaches, which included physical activity components, resulted in a reduction of gestational weight gain and increased the proportion of women without postpartum weight retention [99].

## 8. Comparison of BIA, DEXA, and MRI Methods for Postpartum Women, Including Breastfeeding

In addition to BIA, other methods for assessing body composition include DEXA and MRI, both of which are non-invasive and provide quantitative results. BIA, DEXA, and MRI differ in their mechanisms, precision, availability, costs, and safety [96,101,102,103,104], particularly for postpartum women, including those who are breastfeeding. BIA, using low electrical currents, is considered safe for women in the postpartum period, including breastfeeding mothers. DEXA is not recommended during breastfeeding due to the potential risk of radiation exposure, although it may be used once lactation is complete. MRI is generally safe for breastfeeding women without the use of contrast, though gadolinium contrast can pose potential risks, and its use in lactating women is limited [105].

BIA is portable, inexpensive, and easy to perform, even in non-clinical settings such as at home or in outpatient clinics. In contrast, DEXA and MRI require large, stationary equipment, making them less accessible and more costly. BIA is the least expensive method, while MRI is the most costly, both in terms of equipment and operational expenses.

In terms of accuracy, BIA is less precise due to factors like hydration levels, which can fluctuate postpartum, especially for breastfeeding women. It is primarily used for assessing general body composition. DEXA provides precise measurements of fat, muscle, and bone mass but involves radiation exposure and is not recommended during breastfeeding. MRI offers high precision and spatial analysis of body composition, including fat redistribution and muscle mass changes, which may occur postpartum. However, it requires more complex analysis and is typically used in clinical cases rather than routine assessments.

Clinically, BIA is useful for monitoring general health indicators such as hydration, weight loss, and body composition changes in the postpartum period, including breastfeeding, but it does not provide detailed insights. DEXA offers critical information for metabolic health but is unsuitable during lactation. MRI is useful for complex clinical cases, particularly in assessing fat distribution and muscle condition postpartum, though it is often reserved for specific conditions due to high costs and limited availability.

In summary, BIA is the most suitable method for postpartum women, including those who are breastfeeding, due to its safety, low cost, and accessibility, despite its lower precision. DEXA is unsuitable during breastfeeding due to radiation exposure, while MRI is best reserved for complex clinical cases requiring detailed diagnostics, given its high cost and limited availability (Table 1).

## 9. Limitations of BIA in Postpartum Assessment

Although BIA offers numerous advantages, like any other method, it has several limitations. First and foremost, BIA is highly sensitive to changes in fluid distribution, which can lead to inaccurate estimates of fat mass, lean mass, and hydration status, especially when there is an imbalance between ICW and ECW. This sensitivity makes BIA particularly prone to errors in cases of altered fluid balance, such as in the postpartum period.

Another limitation is the lack of standardization for the postpartum population. The standard equations used in BIA devices to convert impedance measurements into body composition estimates are not optimized for postpartum women. Many of the models are based on healthy adult women of reproductive age but fail to account for the physiological changes that occur during pregnancy and the postpartum period. As a result, these equations may yield inaccurate results, particularly in terms of fat and fat-free mass.

Lactation, which affects metabolism, caloric demand, and fat distribution, can also interfere with the accuracy of BIA results. Enlarged breasts and their altered density due to milk production can influence the segmental body composition analysis, leading to skewed measurements. Furthermore, BIA results can be affected by several external factors, such as the time of day, the timing of the last meal (including salt intake), physical activity prior to measurement, and ambient temperature. Postpartum women may face challenges in adhering to standard measurement protocols, such as fasting or refraining from physical activity before the test, which can complicate the acquisition of reliable results.

Additionally, BIA has limited capacity to detect specific physiological changes, such as the distribution of body fat (e.g., visceral vs. subcutaneous fat), which is critical in evaluating metabolic health after childbirth. It also cannot assess structural changes in muscle and connective tissue, such as pelvic floor muscle weakness. Furthermore, BIA results may vary depending on the manufacturer and model of the device. Portable devices, in particular, tend to be less accurate than more advanced laboratory-based systems.

In summary, while BIA offers many benefits, it has significant limitations in postpartum assessment. Sensitivity to fluid changes, the lack of adapted algorithms, and dependence on measurement conditions represent the main challenges. To increase the clinical value of BIA for this population, further technological advancements, the development of dedicated analysis models, and the use of complementary diagnostic methods in more complex cases are essential.

## 10. Proposals for Practical Application of BIA in Postpartum Women

Performing BIA within 48 h post-partum appears to be a rational strategy. Early post-partum assessment of body composition—prior to discharge from the hospital—may help identify imbalances in fat and lean tissue, which could indicate hidden metabolic risks such as insulin resistance, difficulty with post-partum weight loss, and an increased risk of obesity. Statistically significant differences in several BIA parameters assessed within 48 h after delivery were found when comparing healthy women to those who had been diagnosed with GDM or EGWG during pregnancy [106,107]. Ruszała et al. observed that the fat mass index assessed by BIA within 48 h after delivery was positively correlated with weight retention at 6 months post-partum in both women with EGWG and those with a normal pregnancy course [108].

Early identification of body composition issues allows for timely intervention, potentially preventing the future onset of type 2 diabetes mellitus, obesity, and cardiovascular diseases. BIA can provide valuable information regarding a woman’s nutritional status and overall health, serving as a basis for personalized care plans aimed at optimizing maternal health.

Furthermore, assessing body composition within the first 48 h post-partum can assist healthcare providers in managing weight loss or gain in a safe and gradual manner, which is crucial for women in the early breastfeeding phase. Maintaining optimal body composition in the early post-partum period is associated with better lactation outcomes, as adequate fat stores are important for sustained milk production. Early BIA testing may detect any fluid imbalances or dehydration, which can hinder lactation, allowing healthcare providers to intervene with appropriate hydration strategies. This could be particularly beneficial in identifying women at risk for post-partum depression, as metabolic disturbances may exacerbate mood disorders.

By offering targeted nutritional and lifestyle interventions, BIA results can support breastfeeding women in maintaining an optimal BMI, thereby increasing the likelihood of prolonged breastfeeding. BIA can also help detect changes in muscle mass, which are critical for maintaining overall physical fitness and well-being during the demanding post-partum period. Providing post-partum women with information on their metabolic health can foster a sense of empowerment and encourage proactive health management, improving long-term outcomes.

In summary, integrating BIA into routine post-partum care seems to serve as an early-warning system, enabling healthcare providers to offer timely interventions that support both maternal metabolic health and breastfeeding success.

## 11. Future Research Directions

The advanced version of the traditional BIA method, namely multi-frequency BIA (MF-BIA), holds significant potential, particularly for applications in postpartum health monitoring. This technique allows for more precise measurements by using multiple frequencies to assess different tissues within the body, offering a more comprehensive analysis of body composition [47]. Moreover, MF-BIA can be integrated with mobile devices, enabling women to independently monitor their body composition and track their progress over time [109]. This self-monitoring capability is particularly beneficial in the postpartum period, where women may experience significant fluctuations in body fat, muscle mass, and fluid distribution.

In addition, data from the MF-BIA device can be sent directly to healthcare providers, allowing for remote analysis and the adjustment of recommendations based on the individual’s results. This remote monitoring can help healthcare professionals offer personalized care, ensuring that postpartum women receive timely advice tailored to their specific health status. When combined with modern algorithms, MF-BIA has the potential to provide even more personalized health analyses, offering predictions and guidance that can optimize recovery, weight management, and overall well-being during the postpartum period.

It is suggested that further research be conducted to explore specific directions for the clinical validation of BIA in the management of postpartum women’s health. This includes investigating how clinical data can substantiate the effectiveness of BIA in tracking and managing postpartum changes. Additionally, exploring the combination of advanced technologies, such as multi-frequency BIA, with other diagnostic tools could reveal potential improvements and optimizations for postpartum care. Integrating these innovations may lead to more accurate assessments of metabolic health, hydration status, and body composition, which are critical for enhancing maternal recovery and long-term health outcomes.

## 12. Conclusions

Our study underscores the importance of bioelectrical impedance, particularly during the challenging postpartum period. We have demonstrated that BIA can facilitate the monitoring of body weight by distinguishing between muscle mass, fat mass, and hydration levels. Proper hydration is critical for lactation and ensuring optimal breast milk composition. BIA also enables the assessment of rehabilitation effectiveness in postpartum women. By evaluating body composition, including muscle-to-fat ratio, hydration, and metabolic rate, BIA helps monitor the progression of rehabilitation. It allows for the customization of rehabilitation and dietary plans, which can accelerate recovery and improve overall maternal health post-delivery. Furthermore, BIA aids in the early detection of health issues such as edema, electrolyte imbalances, infections, and preeclampsia. Due to its simplicity and versatility, BIA has the potential to become a gold standard in postpartum care, significantly enhancing the quality of life and health of women during this critical period.

## Figures and Tables

**Table 1:** Comparison of BIA, DEXA, and MRI Methods for Postpartum Women, Including Breastfeeding.

Criteria	BIA	DEXA	MRI
Safety	Safe, no known risks for breastfeeding women	Potentially dangerous due to radiation, limited data on breastfeeding safety	Generally safe, especially without contrast, with precautions for metal implants and pacemakers; gadolinium contrast use is limited during breastfeeding
Availability and logistics	Very easy to achieve; portable and can be performed at home or in various settings	Limited accessibility due to specialized equipment and location requirements	Limited accessibility due to large, stationary equipment and specialized facilities
Costs	Low, affordable equipment and examination costs	Moderate, with high equipment and operational costs	High, both for equipment and operational costs
Precision	Low to moderate precision, affected by hydration levels and other factors	High precision in bone density and body composition measurements	Very high precision, especially in analyzing soft tissues, fat distribution, and muscle mass
Clinical relevance	Moderate, useful for general screening and monitoring hydration and weight changes	High clinical relevance, though caution is advised during breastfeeding	Very high, particularly for complex clinical cases, but rarely used for routine body composition analysis

## Data Availability

No new data were created or analyzed in this study.
